# Metabolic Checkpoints and Lymphoid Neogenesis in Lung Dendritic Cells: Mechanisms Guiding Tolerance and Chronic Lung Inflammation

**DOI:** 10.3390/ijms27062887

**Published:** 2026-03-23

**Authors:** Dara C. Fonseca-Balladares, Gabriela O. S. Costa, Kevin Nolan, Michael H. Lee, Thaís C. F. Menezes, Brian B. Graham, Claudia Mickael

**Affiliations:** 1 Department of Medicine, University of California San Francisco, San Francisco, CA 94158, USA; dara.fonsecaballadares@ucsf.edu (D.C.F.-B.); kevin.nolan@ucsf.edu (K.N.); michael.lee8@ucsf.edu (M.H.L.); brian.graham@ucsf.edu (B.B.G.); 2 Lung Biology Center, Zuckerberg San Francisco General Hospital, San Francisco, CA 94110, USA; 3 Departamento de Medicina, Disciplina de Pneumologia, Universidade Federal de São Paulo (UNIFESP), São Paulo 04021-001, Brazil; siqueira@unifesp.br (G.O.S.C.); thais.menezes@unifesp.br (T.C.F.M.); 4 Division of Pulmonary, Allergy and Critical Care Medicine, Department of Medicine, Anschutz Medical Campus, University of Colorado, Aurora, CO 80045, USA; 5 Cardiovascular Pulmonary Research Laboratories, School of Medicine, University of Colorado, Aurora, CO 80045, USA

**Keywords:** dendritic cells, cDC1, cDC2, lung immunity, immunometabolism, HIF-1α, PD-1/PD-L1, tertiary lymphoid structures, chronic lung disease

## Abstract

Dendritic cells (DCs) are key sentinels in the lung mucosa that interpret environmental signals to either promote tolerance or trigger inflammation, influencing the development of chronic lung diseases. This review highlights recent mechanistic insights showing that metabolic checkpoints serve as upstream regulators of DC fate and activity: inflammatory stimuli activate HIF-1α/mTOR-linked glycolytic pathways that drive maturation, cytokine secretion, antigen presentation, and migration. In contrast, AMPK-related oxidative and lipid metabolism pathways support tolerogenic states that encourage regulatory T-cell responses and inhibit checkpoints like PD-1/PD-L1. We also present evidence that DC subset specialization (cDC1 vs. cDC2) and their tissue location interact with these metabolic pathways to regulate lymphoid tissue formation, including the development and persistence of tertiary lymphoid structures in chronically inflamed lungs. These ectopic lymphoid tissues enhance local immune responses through DC–stromal interactions and ongoing T follicular helper–B cell communication, contributing to persistent inflammation and tissue remodeling in conditions such as COPD, asthma, pulmonary hypertension, and fibrotic interstitial lung disease. Finally, we discuss the translational potential of targeting this immunometabolic–lymphoid pathway, suggesting that modulating metabolic regulators, migratory circuits, and tolerogenic programs could restore immune balance while maintaining host defense—a promising framework for developing advanced therapies for chronic lung inflammation.

## 1. Introduction

**Figure 1:**
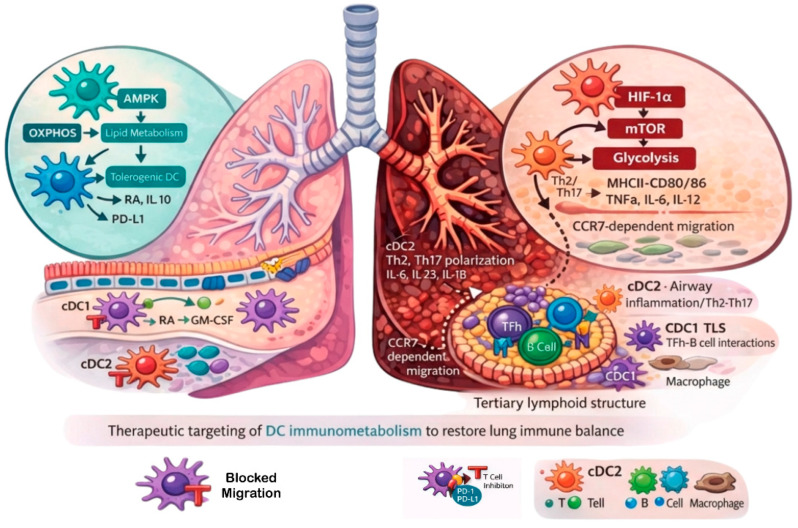
Metabolic programming of dendritic cells regulates lung immune homeostasis and chronic inflammation. Under homeostatic conditions (left), dendritic cells (DCs) predominantly utilize AMPK-driven oxidative phosphorylation (OXPHOS) and lipid metabolism, promoting a tolerogenic phenotype characterized by retinoic acid (RA) and IL-10 production and PD-L1 expression. Conventional DC subsets (cDC1 and cDC2) support balanced immune surveillance and maintain tissue homeostasis. During chronic inflammation (right), DCs shift toward HIF-1α/mTOR–dependent glycolysis, resulting in an immunogenic phenotype with increased MHC-II, CD80/CD86, and pro-inflammatory cytokines (IL-6, IL-23, IL-12, TNFα). Inflammatory cDC2 promote Th2 and Th17 polarization, while cDC1 contribute within tertiary lymphoid structures to enhanced Tfh–B cell interactions. Dysregulated DC activation increases CCR7-dependent migration and amplifies pathogenic T cell responses.

Dendritic cells (DCs) are specialized antigen-presenting cells that connect innate and adaptive immunity, playing a key role in maintaining immune balance within the lung’s constantly exposed mucosal environment [1,2,3,4,5,6]. There are two conventional/classical DC subtypes in the lung: cDC1 and cDC2, as well as plasmacytoid DCs (pDCs) and monocytic-derived DCs (moDCs) [3,6,7]. Disruption of DC-mediated tolerance can lead to chronic lung diseases such as asthma, COPD, idiopathic pulmonary fibrosis (IPF), and pulmonary arterial hypertension (PAH), in which abnormal activation and migration promote ongoing inflammation and tissue remodeling [8,9,10,11,12,13,14]. Recent research emphasizes the importance of DC immunometabolic reprogramming (via HIF-1α, mTOR, and AMPK pathways) and their role in forming tertiary lymphoid organs (TLOs), which serve as central mechanisms that boost local adaptive immunity (Figure 1) [15,16,17,18,19,20]. These findings suggest that DCs are promising targets for therapy—through metabolic adjustments, checkpoint modulation, and tolerogenic approaches—to restore immune balance and prevent tissue damage in chronic lung diseases [21,22,23,24].

## 2. The Lung DC Network: Subsets, Origins, and Spatial Niches

Dendritic cells (DCs) arise from hematopoietic stem cells through common DC progenitors (CDPs) and pre-DCs, which generate the conventional DC subsets cDC1 and cDC2 [25,26,27,28]. cDC1 fate is sustained by IRF8, BATF3, and ID2, whereas cDC2 development is driven by IRF4 with contributions from Notch2, RelB, and KLF4 [29,30,31,32,33,34]. IRF4 deficiency causes combined immunodeficiency with marked defects in T helper differentiation and DC-related myeloid differentiation [35].

In humans, cDC1s are marked by XCR1 and CLEC9A expression; cDC2s by CD1c (BDCA-1) and SIRPα. In mice, cDC1s are enriched for CD103, whereas cDC2s are enriched for CD11b and CD301b [36,37,38,39,40,41,42]. Although these markers differ across species, they delineate broadly conserved functional programs: human and mouse cDC1s both excel at cross-presentation and induction of cytotoxic CD8^+^ T-cell responses, while cDC2s in both species preferentially drive CD4^+^ T-cell polarization toward Th2 and Th17 pathways. However, some functional divergence exists—certain human cDC2 subsets display broader heterogeneity than their murine counterparts, and not all marker-defined populations have perfect one-to-one equivalence (Table 1). Therefore, while phenotypic markers differ between species, the underlying functional specializations of cDC1 and cDC2 lineages are largely conserved, allowing cross-species comparisons with attention to these caveats.

In the pulmonary environment, cDC1s are located near epithelial surfaces where they efficiently capture cell-associated antigens [43,44,45]. On the other hand, cDC2s, reside predominantly in the lung interstitium, sampling soluble antigens and interacting with stromal and immune cells [46,47].

These subsets exhibit strikingly divergent roles. cDC1s excel at cross-presentation, delivering exogenous antigens on MHC-I to prime CD8^+^ T cells and promote Th1 polarization, a function critical for antiviral and antitumor immunity [48,49]. In contrast, cDC2s preferentially present antigens on MHC-II, driving CD4^+^ T cell responses and promoting Th2 and Th17 differentiation, which are pathways strongly implicated in allergic and fibrotic lung disease [3,46,49]. Notably, there is evidence of increased density of cDC2s in the lung tissue of humans with pulmonary arterial hypertension (PAH). In experimental hypoxia-induced PH, perivascular cDC2s increase in numbers, and depleting them using CD301b-DTR mice results in protection from the PH phenotype, including a reduction in vascular inflammation and remodeling [42]. Critically, cDC depletion in hypoxia-induced PH (HPH) models resulted in the widespread downregulation of numerous cytokines, chemokines such as CCL17/TARC, CCL19/MIP-3β, CCL22/MDC), and growth factors [42]. cDC2s also contribute to schistosomiasis-induced PH [50]. Interestingly, depletion of cDC1s using Batf3^-/-^ mice causes more severe experimental PH, through as-yet unclear mechanisms [50]. In a model of liver-induced schistosomiasis injury, depletion of cDC1s worsens the liver injury with more severe Th2 immunity [51].

Although cDC1 and cDC2 are the main antigen-presenting cells in the lung, other dendritic cell subsets, such as plasmacytoid DCs (pDCs) and monocyte-derived DCs (moDCs), also have vital roles in influencing immune responses during infection, tolerance, and chronic inflammation. pDCs are unique in their ability to produce large quantities of type I and III interferons (IFNs) upon viral recognition, thereby orchestrating antiviral defense and bridging innate and adaptive immunity [52,53]. IFN-I signaling enhances NK cell cytotoxicity, CD8^+^ T-cell responses, and B-cell antibody production, positioning pDCs as key regulators of immune responses during respiratory viral infections. In homeostasis, pDCs contribute to peripheral tolerance by transporting harmless antigens to lymph nodes and inducing regulatory pathways, including IL-10 and IDO (Indoleamine 2,3-dioxygenase)-mediated suppression of effector responses [54,55]. These dual roles, antiviral defense and tolerance, underscore pDC plasticity in lung immunity.

On the other hand, moDCs arise during inflammation and complement conventional DC networks by amplifying cytokine responses and promoting T helper polarization [10]. In acute viral infections, TLR3-activated moDCs trigger epithelial IL-33 release, linking innate sensing to chronic type 2 inflammation and airway remodeling [56]. This capacity to sustain pathogenic immune circuits positions moDCs as potential drivers of disease progression in asthma and COPD.

Both pDCs and moDCs interact with cDC subsets to fine-tune lung immunity. In chronic lung disease, moDC-driven cytokine storms and pDC dysfunction can exacerbate inflammation or impair tolerance, contributing to heterogeneous disease phenotypes.

Growing evidence indicates that distinct dendritic-cell subsets exhibit intrinsic metabolic specializations that shape their immunogenic or tolerogenic tendencies. cDC1s, which excel at cross-presentation, frequently engage oxidative metabolism pathways and maintain higher basal mitochondrial function, consistent with their reliance on sustained antigen processing and cross-presentation programs—a pattern supported by studies demonstrating OXPHOS-linked regulatory circuits in tolerogenic and steady-state DCs [57,58]. In contrast, cDC2s, which preferentially drive Th2 and Th17 responses, show stronger coupling to glycolytic activation under inflammatory conditions, including TLR- and hypoxia-driven pathways that upregulate HIF-1α/mTOR signaling and enhance their cytokine production and migration [15,59].

pDCs, which coordinate antiviral responses, rely heavily on type I/III interferon production and appear metabolically distinct, engaging lipid-modifying pathways and redox-balancing circuits that support interferon output and tolerance induction [52,55]. Meanwhile, moDCs, generated during inflammation, adopt a metabolism characteristic of activated monocyte-derived cells—showing rapid glycolytic induction and nitric-oxide–linked suppression of mitochondrial respiration, supporting their role in amplifying inflammatory cytokine networks [10,56].

Together, these findings demonstrate that metabolic checkpoint engagement is not uniform across DC subsets: rather, it is shaped by ontogeny, niche cues, and functional specialization. Recognizing this heterogeneity strengthens the link between metabolic state and subset-specific contributions to chronic lung inflammation, particularly in diseases where cDC2-driven Th2/Th17 programming or pDC dysfunction is predominant.

## 3. The Lung as an Immunological Interface

The lungs’ primary role in gas exchange places them in constant contact with the external environment, exposing them to inhaled antigens such as pathogens, allergens, and particulate matter [60]. To maintain homeostasis, the lung must balance this physiological function with robust immune surveillance and defense [61,62].

This equilibrium relies on multiple layers of protection. Physical and chemical barriers—tight epithelial junctions, mucociliary clearance, antimicrobial peptides, and secreted immunoglobulin A form the first line of defense [60]. Beyond these barriers, dendritic cells (DCs) are the principal immune sentinels of the lung [8,62,63]. Strategically positioned throughout the airway mucosa and parenchyma, DCs continuously sample inhaled material and integrate signals from the local microenvironment to orchestrate adaptive immunity [53,64].

After capturing antigens, DCs migrate to draining bronchial lymph nodes, where they present processed peptides on MHC molecules to the T cell receptors (TCRs) of naïve T cells [53]. This interaction determines whether the immune system activates or tolerates the antigen, a critical decision for preventing excessive inflammation and tissue damage [3,8]. Through this function, DCs maintain the delicate balance between tolerance and immunity that is essential for preserving lung integrity [65].

## 4. From Sensing to Positioning: PRR Activation and Trafficking Programs

During pulmonary inflammation, DCs act as first responders by sensing pathogens and allergens via pattern recognition receptors (PRRs), including Toll-like receptors (TLRs) and NOD-like receptors (NLRs) [66,67]. Pro-inflammatory cytokines such as IL-1β, TNF-α, and GM-CSF promote DC maturation (also called activation), while the anti-inflammatory cytokine IL-10 induces tolerance [68,69]. After DC maturation, they upregulate MHC-II and co-stimulatory molecules (CD80/CD86) through NF-κB and IRF pathways [70,71]. Mature DCs secrete proinflammatory cytokines such as TNF-α, IL-6, and IL-12, which shape the early immune environment [1,66,72,73]. DC activity is profoundly shaped by Toll-like receptor (TLR) signaling, the core innate sensing mechanism, which activates transcription factors like NF-κB and interferon regulatory factors (IRFs) [68,74]. The pathways frequently emphasize dual roles for signaling molecules in activating both the NF-κB and IRF pathways [74]. For instance, the TLR3 and TLR4 signaling axis mediates slow activation of NF-κB and induction of co-stimulatory molecules [74]. TLR stimulation also drives rapid metabolic reprogramming in DCs, a switch toward glycolysis that is required for full maturation and cytokine output [75]. Beyond TLRs, inflammasome activation via NLRs further amplifies IL-1β and IL-18 production during bacterial and viral infections [76,77,78]. Activated DCs migrate to draining mediastinal lymph nodes via the CCR7–CCL19/CCL21 axis, enabling antigen presentation and T cell priming [79,80,81], which is further modulated by epigenetic control and prostaglandin E_2_-dependent signal transduction [82,83,84,85]. By enabling responsiveness to CCL19/CCL21 gradients, CCR7 selectively permits activated cDCs to migrate to lymph nodes, while CCR7^−^ cDCs remain tissue resident. Foundational work also showed CCR7 upregulation on mature DCs and chemotaxis to CCL19/CCL21, with PGE_2_ facilitating CCR7 signal transduction [86]. In disease, elevated CCR7 ligands in eosinophilic pneumonia suggest that this axis contributes to DC accumulation in the inflamed lungs [87]. Notably, CCR7 deficiency in hematopoietic cells enhanced fungal clearance in pulmonary aspergillosis, illustrating the context-dependent roles of this migratory program [88].

## 5. Metabolic Checkpoints Governing DC Fate

Although the term “checkpoint” is broadly used in immunology, here we define a metabolic checkpoint as a regulatory metabolic node that determines whether dendritic cells (DCs) adopt an immunogenic or tolerogenic fate in response to environmental cues. Such checkpoints integrate signals from nutrient availability, oxygen tension, and pattern-recognition receptors to enforce distinct metabolic programs [17,89]. In inflammatory settings, activation of the HIF-1α/mTOR axis shifts DCs toward glycolysis, supporting maturation, cytokine production, antigen presentation, and migration [15,75,90]. Conversely, activation of AMPK-dependent oxidative phosphorylation and fatty-acid oxidation promotes tolerogenic features, including IL-10 production, retinoic-acid–driven Treg induction, and PD-L1 expression [57,58].

Thus, in this review, a metabolic checkpoint refers to a metabolically controlled decision point that commits DCs toward either immunogenic activation or tolerance, providing a mechanistic bridge between environmental sensing and functional output [15,59].

Both cDC1s and cDC2s undergo rapid and functionally important metabolic reprogramming in response to the lung microenvironment [89] and upon activation. Inflammatory stimuli and hypoxia shift the cellular metabolism of DCs toward aerobic glycolysis, a metabolic reprogramming that supplies biosynthetic intermediates required for membrane biogenesis, cytokine production, and the energetic demands of migration and antigen presentation [81]. Much of the mechanistic understanding of TLR-driven glycolysis—including rapid induction of glycolytic flux through HIF-1α and mTOR—derives from in vitro studies using bone-marrow–derived DCs (BMDCs) [15,75].

However, glycolytic programming has also been demonstrated in vivo in the lung, where airway exposure to allergens or pathogens activates TLR pathways that enhance glycolysis in airway-draining DCs [15]. The switch to increased glycolysis is coordinated largely by hypoxia-inducible factor-1α (HIF-1α) and mTOR signaling [90,91]. HIF1a causes glycolysis upregulation, including by increasing expression of PFKFB2, resulting in the synthesis of fructose-2,6-bisphosphate which allosterically increases the activity of two key glycolytic enzymes, phosphofructokinase 1 (PFK-1) and fructose 1,6-bisphosphatase (FBPase-1) [92]. Hypoxia activates HIF-1α, promoting glycolysis and enhancing DC migration. Glycolytic reprogramming is necessary for DC migration to draining lymph nodes. This has been demonstrated mechanistically in in vitro systems (TLR-stimulated DCs showing glycolysis-dependent CCR7 signaling) [75] and in vivo, where hypoxia-exposed DCs show HIF-1α–dependent migratory enhancement in mouse lung models [59]. HIF-1α–dependent metabolic changes have been shown in vitro using cultured DCs [59] and in vivo in lung tissue—for example, in hypoxia-induced pulmonary hypertension and airway inflammation models showing increased DC migration and activation [15,42]. Experimentally, blockade of glycolysis impairs the maturation and T-cell stimulatory capacity of DCs in a model of rheumatoid arthritis [93]. On the other hand, mTOR integrates environmental cues to promote glycolytic reprogramming and full DC maturation. Most mechanistic studies of mTOR-dependent DC activation employ in vitro BMDC systems [90], but mTOR activity is also implicated in vivo in lung inflammation, where mTOR-driven glycolysis enhances DC capacity for cytokine production and migration. Evidence in lung models comes from studies using TLR activation or hypoxia to induce glycolytic shifts.

Conversely, AMPK and fatty acid oxidation are enriched in tolerogenic DCs (tDCs)--cells with increased capacity to induce anti-inflammatory regulatory T cells (Tregs) [58]. The detailed metabolic mechanisms governing AMPK-mediated tolerogenesis (e.g., induction of RALDH^+^ DCs) are primarily derived from in vitro BMDC or human monocyte-derived DC experiments [57,58,94]. In the lung in vivo, tolerogenic DCs have been observed during radiation-induced injury and fibrotic processes, but the specific AMPK-dependent metabolic switches have not yet been dissected at the same mechanistic level [95]. Thus, AMPK-linked tolerogenic programming remains supported by strong in vitro mechanisms and in vivo correlative evidence. In cell culture experiments, activating AMPK induces tDCs, whereas blocking AMPK and fatty acid oxidation suppresses tDCs [58]. Together, these data suggest DC metabolism may be targetable to modulate chronic inflammation. One specific example is pharmacologically activating AMPK, such as with the medications metformin or rosiglitazone, which are FDA-approved for treatment of diabetes through multiple mechanisms including increasing insulin sensitivity.

While many principles of dendritic cell (DC) immunometabolism are conserved across tissues, it is important to distinguish findings demonstrated in vivo in the lung from those derived primarily from in vitro systems or extrapolated from other organs. In the lung, several metabolic mechanisms have been validated directly in vivo: hypoxia-induced activation of HIF-1α, which enhances DC glycolysis and migration, has been shown in murine models of pulmonary inflammation [59]; PRR-driven glycolytic reprogramming has likewise been demonstrated in airway-draining DCs responding to inhaled allergens or pathogens [15,75]. Additionally, lung-resident cDC2 populations have been shown in vivo to adopt glycolytic, pro-inflammatory phenotypes in COPD, asthma, and pulmonary hypertension, linking metabolic activation to disease-relevant Th2/Th17 or Tfh induction [42,46,96].

In contrast, several mechanistic insights into AMPK-dependent oxidative metabolism, fatty-acid oxidation, and tolerogenic DC programming come from controlled in vitro or ex vivo systems. Studies demonstrating AMPK-mediated induction of RALDH^+^ tolerogenic DCs or vitamin D–driven tolerogenic differentiation were largely performed in cultured bone marrow–derived or monocyte–derived DCs [57,58,97]. These in vitro findings align conceptually with observations that tolerogenic DCs appear in vivo during lung injury or fibrosis, but the specific metabolic switches governing their emergence in lung tissue remain less well defined.

Thus, while glycolytic activation programs (via HIF-1α/mTOR and PRR signaling) are supported by robust in vivo lung evidence, tolerogenic metabolic programs (via AMPK and lipid oxidation) rely more heavily on in vitro mechanistic studies, with limited in vivo correlative findings. Clarifying these distinctions underscores both the strength of current data and the need for future lung-specific metabolic tracing studies.

The lung microenvironment imposes unique metabolic pressures that shape dendritic-cell fate and activation. Hypoxia, which arises in chronically inflamed or remodeled lung tissue, stabilizes HIF-1α and drives a glycolytic metabolic checkpoint that promotes DC maturation, cytokine production, and migration, which has been demonstrated both in vitro and in vivo [42,59]. In contrast, the steady-state lung is enriched in surfactant-derived lipids, which support oxidative and lipid-metabolism programs associated with tolerogenic DC states [94]. AMPK-linked pathways and fatty-acid oxidation—largely defined through in vitro DC studies—align with this lipid-rich environment and help maintain regulatory homeostasis at mucosal surfaces [57,58]. Together, these features of the pulmonary niche create a metabolic landscape in which fluctuating oxygen tension and abundant lipids selectively engage glycolytic or oxidative checkpoints. This dynamic tuning enables lung-resident DCs to shift between immunogenic and tolerogenic programs depending on the environmental context, thereby linking lung physiology directly to DC metabolic specialization. In addition to oxygen and lipid availability, emerging work shows that lactate-rich inflamed tissues can activate the lipid-anabolic transcription factor SREBP2, driving the differentiation of metabolically reprogrammed regulatory DCs (mregDCs), a mechanism that—while defined in tumor models—highlights how microenvironmental metabolites may similarly shape DC metabolic checkpoints in the lung [98].

Emerging evidence indicates that dendritic cells exhibit substantial metabolic plasticity, enabling them to transition between glycolytic, oxidative, and lipid-dependent metabolic programs in response to environmental cues. This flexibility is apparent across multiple DC subsets. For example, inflammatory stimuli and hypoxia can rapidly shift both cDC1 and cDC2 toward HIF-1α– and mTOR-dependent glycolysis, thereby enhancing antigen presentation, cytokine production, and migratory capacity [15,59]. Conversely, engagement of AMPK and fatty-acid oxidation pathways enables DCs to adopt tolerogenic phenotypes, driving Treg induction and limiting effector T-cell activation—programs largely defined in vitro but consistent with tolerogenic DC states observed in vivo during lung injury and fibrotic remodeling [57,58,95].

Plasticity is also reflected in the capacity of certain DC subsets to acquire metabolic features typically associated with other DC subsets. In inflammatory environments, cDC2 can adopt metabolic traits resembling those of activated cDC1—such as increased glycolysis—especially within tertiary lymphoid structures in COPD and pulmonary hypertension, where ongoing antigen presentation demands high energetic support [96]. Conversely, under homeostatic or regulatory cues, even typically immunogenic DCs can shift toward oxidative and lipid-metabolic programs that promote tolerance, as seen in pDCs that transport innocuous antigens to lymph nodes and inducing IL-10 or IDO [52,55].

This capacity for bidirectional metabolic reprogramming has direct implications for chronic lung inflammation. In diseases such as COPD, asthma, pulmonary hypertension, and fibrotic ILDs, sustained exposure to hypoxia, altered lipid environments, cytokines, or repeated PRR stimulation may continuously push DCs toward glycolytic activation, reinforcing pathogenic Th2, Th17, and Tfh pathways. Conversely, the failure to appropriately activate AMPK-linked tolerogenic circuits may contribute to impaired checkpoint restraint, loss of Treg control, and chronic inflammatory amplification. Recognizing DC metabolic plasticity underscores the need for therapeutic strategies to account not only for basal DC metabolic wiring but also for dynamic environmental signals in the lung that dictate whether DCs adopt immunogenic or tolerogenic roles.

## 6. Lymphoid Neogenesis and TLS/TLO Formation

Chronic lung inflammation leads to the development of tertiary lymphoid organs (TLOs), also known as tertiary lymphoid structures (TLSs), in adjacent non-lymphoid tissues near airways and blood vessels. These ectopic immune cell clusters form through coordinated interactions among immune cells, such as Th17 cells, T follicular helper cells, B cells, and dendritic cells, as well as stromal fibroblasts and local chemokines and cytokines like CXCL13, CCL19, CCL21, IL-17, and lymphotoxin [96,99,100,101]. Similar in structure and function to secondary lymphoid organs, TLOs feature distinct T and B cell zones, follicular dendritic cell networks, and high endothelial venules that facilitate lymphocyte recruitment, antigen presentation, and adaptive immune responses at the local level [91,102,103,104,105,106]. In TLOs, dendritic and other antigen-presenting cells process tissue-derived antigens for T cell activation and differentiation [102,105,106,107]. Germinal center–like areas support B cell proliferation, somatic hypermutation, and affinity maturation. These ongoing processes sustain chronic immune activation in lung diseases such as COPD and fibrosis and may generate high-affinity antibodies, including autoantibodies [103,106,108,109,110], that can spread and contribute to systemic autoimmunity [103,106,108,109].

Dendritic cells (DCs) play a central role in initiating tertiary lymphoid organ (TLO) formation by secreting chemokines like CXCL13 and CCL20, which attract follicular T helper (Tfh) cells and B cells [111]. Guillaume et al. (2025) emphasize the interactive signaling between DCs and stromal cells in TLO development, driven by chemokines that direct lymphocyte positioning—specifically, CXCL13 guides B cells and Tfh cells [102]. Calvanese et al. (2024) show that prolonged type I interferon signaling promotes lymphotoxin production in lymphoid cells, which in turn stimulates stromal cells to produce CXCL13, aiding germinal center formation [99]. Similarly, Durand et al. (2019) indicate that human cDC2 cells strongly induce Tfh cell polarization, and Tfh cells themselves produce CXCL13, enhancing B cell recruitment and follicle development [112]. Tfh cells, which are antigen-experienced CD4^+^ T cells, upon recognizing their specific antigen via DCs, promote germinal center formation to facilitate B cell maturation and activation [113]. Collectively, these studies highlight the importance of DC–stromal–Tfh interactions in chemokine-driven TLO development and function during chronic inflammation.

TLO formation is highly relevant in COPD, idiopathic pulmonary fibrosis (IPF), pulmonary hypertension (PH), and severe asthma, where it reflects persistent immune activation and contributes to disease progression. In COPD, TLOs, often termed inducible bronchus-associated lymphoid tissue (iBALT), form near airways and vessels and correlate with severe airflow limitation. cDC2s are central to this process: they accumulate in the lung, express OX40L, and induce IL-21^+^CXCL13^+^ Tfh-like cells, which promote B cell recruitment and TLO organization. This cDC2-driven TLO formation sustains local adaptive immunity, autoantibody production, and chronic inflammation [96,114,115,116,117]. In IPF, conventional DCs accumulate in fibrotic regions and within lymphoid follicles, thereby bridging innate and adaptive immunity and supporting the organization of ectopic lymphoid structures. Their presence in TLOs correlates with ongoing immune activation and may influence prognosis [118,119,120]. In pulmonary arterial hypertension, TLOs are observed around remodeled pulmonary vessels and are associated with CXCL13 expression, local B and T cell activation, and in situ antibody production, supporting a role for adaptive immune responses in sustaining vascular inflammation and disease progression [13,121]. TLOs are less well characterized in severe asthma but occur in patients with persistent or neutrophilic disease. Therefore, lung DCs promote local lymphoid neogenesis and an imbalance between tolerance and inflammation, contributing to disease heterogeneity [114,122,123].

## 7. Tolerance Circuits and Checkpoint Restraint

Dendritic cells (DCs) are not only sentinels of immunity but also guardians of tolerance. In the lung, where continuous exposure to environmental antigens requires restraint, DCs prevent harmful inflammation by promoting regulatory pathways under steady-state conditions [89]. This homeostatic function is essential to avoid chronic inflammatory diseases while preserving the ability to mount protective responses. Although the terms “tolerance” and “anti-inflammatory responses” are sometimes used interchangeably, they represent related but distinct immunological states. Tolerance refers to an active, antigen-specific regulatory program in which dendritic cells (DCs) promote long-term restraint of immune activation—primarily through induction of Foxp3^+^ Tregs, PD-1/PD-L1 signaling, and presentation of antigens in a low-costimulatory context. Anti-inflammatory responses, in contrast, are broader and often transient mechanisms that suppress ongoing inflammation—for example, IL-10-mediated feedback or arrest of DC maturation—without necessarily establishing sustained antigen-specific unresponsiveness. In the lung, where continuous exposure to inhaled antigens demands precise calibration, DCs can participate in both processes: tolerogenic DCs enforce antigen-specific immune restraint to prevent chronic inflammation, while anti-inflammatory circuits act as rapid-response brakes that limit tissue damage during ongoing immune activation. Clarifying this distinction underscores how metabolic and checkpoint pathways differentially shape DC-mediated tolerance versus more generalized inflammation-resolving programs.

Mechanisms of tolerance induction:

Immature DCs, characterized by low expression of MHC-II and co-stimulatory molecules, present antigens in a non-inflammatory context, favoring tolerance over immunity [124]. Three complementary mechanisms underpin this process:

Regulatory T cell (Treg) induction: DCs produce IL-10 and TGF-β and express PD-L1/PD-L2, driving Foxp3^+^ Treg differentiation [125,126,127].

Anergy or deletion of autoreactive T cells: Antigen presentation without co-stimulation, reinforced by PD-1:PD-L1 signaling, suppresses effector activation [128,129].

Negative feedback regulation: IL-10 from Tregs arrests DC maturation, maintaining an anti-inflammatory state [130].

The PD-1:PD-L1 axis is a cornerstone of peripheral tolerance, limiting T-cell proliferation and cytokine secretion to prevent tissue damage [131,132,133]. Recent studies highlight the bidirectional interaction between DCs and Tregs via AHR (aryl hydrocarbon receptor) signaling. In this process, DCs encourage Treg expansion through IL-10 and TGF-β pathways, while Tregs in turn suppress DC activation. This reciprocal communication creates a circuit that maintains immune tolerance during chronic lung inflammation [99].

Certain dendritic cell subsets play specialized roles in maintaining tolerance within the lung. CD103^+^ cDC1s promote mucosal homeostasis by inducing Foxp3^+^ regulatory T cells through retinoic acid and TGF-β signaling, ensuring tolerance to inhaled antigens [134,135,136]. Plasmacytoid DCs (pDCs) complement this function by transporting harmless antigens to lymph nodes and suppressing allergic sensitization via IL-10 and IDO pathways [54,55]. In chronic obstructive pulmonary disease (COPD), cDC2s expressing PD-L1 exhibit enhanced tolerogenic activity, inducing Tregs and producing IL-10 and TGF-β1 [137]. Notably, recent clinical data reveal that reduced PD-L1^+^ cDC1 in COPD correlates with rapid functional decline and Th17-skewed inflammation, implicating checkpoint failure as a key driver of tolerance breakdown [138].

Tolerogenic DCs (tDCs) also emerge during lung injury and fibrosis. In radiation-induced lung injury, tDCs interact with Tregs and regulatory B cells to produce IL-10, IDO (indoleamine 2,3-dioxygenase), and TGF-β, thereby dampening inflammation and potentially limiting fibrotic progression [95]. These findings underscore DC plasticity and suggest that enhancing tolerogenic programs could mitigate chronic lung remodeling.

Loss of DC-mediated tolerance, through reduced PD-L1, altered cytokine signaling, or environmental insults, contributes to chronic inflammation in asthma and COPD [139,140]. Cigarette smoke disrupts tolerogenic pathways, recruiting pDCs and promoting pathogenic responses [141,142]. The PD-L1^+^ cDC1 deficit in rapid-decliner COPD further supports a model where checkpoint insufficiency drives Th17-skewed inflammation and accelerated disease progression [138].

In summary, DCs maintain lung homeostasis by inducing Tregs, enforcing inhibitory checkpoints, and sustaining an immature phenotype under steady-state conditions. New evidence links PD-L1 loss on cDC1 to rapid COPD progression, while tolerogenic DC programs in lung injury and ex vivo DC therapies offer promising avenues to restore immune balance. These insights position DCs as both guardians and therapeutic targets in chronic lung disease.

## 8. Coupling Metabolism, TLS, and Tolerance

The specialization of conventional DC subsets provides a mechanistic scaffold for these divergent outcomes: cDC1, guided by IRF8/BATF3 programs, excels at cross-presentation and CD8^+^ T-cell priming, which are essential for antiviral and antitumor defense, whereas cDC2, under IRF4/Notch2/RelB/KLF4 control, preferentially drives CD4^+^ T-cell polarization toward Th2 and Th17 pathways [143,144,145]. In chronic disease, this balance tilts. Failures of DC-mediated tolerance, exemplified by diminished PD-L1^+^ cDC1 populations in COPD, permit sustained effector activity, while cDC2-biased activation fuels persistent inflammation, tissue remodeling, and the emergence of tertiary lymphoid organs that perpetuate local adaptive immunity through ongoing interactions with Tfh and B cells [96,146]. Layered atop subset identity, immunometabolic wiring imposes powerful control over DC fate: PRR-driven activation engages HIF-1α and mTOR to shift DCs toward glycolysis, enabling full maturation, cytokine production, and migration, whereas AMPK activation favors fatty-acid oxidation and RALDH^+^ tolerogenic programs that expand regulatory T cells and help reestablish immune restraint [15,17,57].

## 9. DCs in Chronic Lung Diseases

Because the lung is a large mucosal surface that directly interacts with the external environment, the sensing and communication functions of DCs are highly relevant to a number of chronic lung diseases. In COPD, human lung DCs can be detected by scRNAseq, and isolated by flow cytometry. Among the DC subtypes from COPD patients, cDC2s are the most effective subset for activating Tfh cells [96]. In animal models of asthma, cDC2s specifically drive Th2 inflammation and airway remodeling, and their depletion abrogates pathology [46,146]. cDC2s are the major cells that promote Tfh differentiation in asthma [147].

In fibrotic ILDs such as IPF, immature DCs appear adjacent to areas of epithelial hyperplasia and fibrosis, while mature DCs are present in lymphoid-follicle-like aggregates with T and B cells [118]. In the peripheral blood of untreated IPF patients, there is a reduction in the number of DCs, and lower levels of cDC2s specifically correlate with worse prognosis [148]. Communication between DCs and fibroblasts via TLR9–AHR signaling promotes profibrotic programs [149].

Overall, dendritic cells emerge as key intermediaries linking environmental sensing to adaptive immunity and tissue remodeling in chronic lung diseases.

## 10. Therapeutic Implications

Based on their myriad roles in lung biology, DCs are emerging as promising therapeutic targets in lung diseases. One topic of particular interest is tolerogenic DCs (tDCs). While less clearly defined in the lungs, their purported roles in autoimmune diseases and organ transplantation suggest that the induction of tDCs should be explored as a therapeutic strategy against various lung diseases with inflammatory pathobiology. For instance, metabolic modulation with agents like the AMP-activated kinase activator metformin [58,150,151], vitamin D [97,152], rapamycin [153,154], and dexamethasone [58,155] is known to induce tDCs, thereby resulting in immune tolerance and suppression of inflammation. Pharmacologic activation of AMPK (e.g., metformin, rosiglitazone) or inhibition of mTOR has been shown to shift DC metabolism toward tolerogenic programs. These findings are largely based on in vitro DC conditioning [58,97], while the effects of metabolic modulators in vivo in lung disease remain supported primarily by correlative findings in asthma, COPD, and pulmonary hypertension models. Therapeutic strategies to restore immune tolerance in chronic lung disease increasingly focus on dendritic cell (DC) reprogramming. Emerging translational approaches now offer tangible strategies to reprogram dendritic cells (DCs) for clinical benefit in chronic lung disease. One avenue involves pharmacologic metabolic modulation, in which agents such as metformin, rapamycin, vitamin D, or AMPK activators induce tolerogenic DC programs by shifting metabolism toward oxidative phosphorylation and fatty acid oxidation. Another developing strategy is ex vivo generation of tolerogenic DCs, where patient-derived monocytes are differentiated into metabolically conditioned tDCs and re-administered to suppress pathogenic T-cell responses—an approach already tested in preclinical models of asthma and COPD with encouraging results. Additional precision methods include receptor-targeted delivery systems, such as antibodies or nanoparticles directed to DC-enriched endocytic receptors (e.g., DEC-205, CLEC9A), enabling subset-specific delivery of antigens or immunoregulatory cargo while minimizing systemic exposure. Finally, lung-targeted nanoformulations, including intranasal PLGA-based particles, can concentrate metabolic modulators or tolerogenic cues within the airways and draining lymphoid tissues. Together, these mechanistically grounded strategies provide a feasible roadmap for clinically reprogramming DCs to restore immune balance while preserving host defense. Beyond pharmacologic modulation, ex vivo–generated tolerogenic DC therapies have demonstrated promise in preclinical models of asthma and COPD, where they suppress effector T-cell responses and expand Tregs, highlighting their translational potential for restoring tolerance and mitigating chronic inflammation in the lung [156].

DCs may be additionally harnessed to suppress inflammation via other mechanisms. For example, N-acetylcysteine [157,158] and heme oxygenase-1 [159] can dampen DC activation and subsequent inflammation. Collectively, these aspects of DC biology hold promise for treating chronic inflammatory and fibrotic lung diseases, restoring immune balance, and limiting tissue remodeling.

A growing body of evidence supports DC regulation as a potential therapeutic approach specifically in managing pulmonary conditions, including pulmonary fibrosis [160,161] and pulmonary hypertension [42]. Key questions remain, however, as the field explores clinically targeting DCs. Beyond tDC approaches, subset-precision modulation in the lung can be pursued by altering conventional DC abundance—for example, FLT3 ligand–based strategies expand circulating and tissue DC pools in vivo and provide a controllable lever to increase cDC lineages when the goal is to enhance protective antigen presentation [162]. Conversely, proof-of-concept depletion/perturbation studies in pulmonary inflammation models indicate that selectively reducing pathogenic cDC2 activity can attenuate remodeling, motivating translational surrogates (subset-directed blocking antibodies or targeted delivery platforms) rather than genetic tools [163]. A practical lung-relevant route to skew DC function without whole depletion is receptor-targeted delivery of antigen and/or immunomodulators to endocytic receptors enriched on DC subsets—using antibody–cargo constructs that enhance uptake and presentation in the intended subset (e.g., CLEC9A for cDC1-biased delivery and DEC-205 for broader cDC targeting), with conditioning cues determining whether the outcome is immunogenic or tolerogenic [164]. Importantly for respiratory diseases, the route of administration can provide an additional layer of specificity: intranasal or inhaled nanoformulations (including PLGA-based and other pulmonary delivery systems) can enrich payload exposure in airways and lung-draining lymphoid sites while limiting systemic immunomodulation [165]. In established chronic lung inflammation, interrupting co-stimulatory nodes that sustain DC–Tfh coupling (e.g., the OX40L–OX40 axis implicated in lung cDC2-driven Tfh-like programs and lymphoid neogenesis) provides a mechanistically grounded means to blunt TLS/TLO reinforcement rather than broadly suppressing immunity [96]. Together, combining (i) controlled expansion/conditioning (e.g., FLT3L and metabolic or tolerogenic programming), (ii) subset-restricted receptor targeting, and (iii) niche-focused interruption of TLS-supporting signals provides a feasible roadmap to dampen pathogenic lung circuits while preserving host defense.

Careful subset- and timing-specific studies are needed, as DCs can both promote and dampen inflammation, and metabolic interventions may differentially affect migration, antigen presentation, and tolerogenic programming across cDC1 and cDC2. As each DC subset may acquire features of other subsets during disease states [31,166,167,168], a careful dissection of the individual and combined roles of DC subpopulations is warranted. Whether tDC-mediated immune tolerance confers unique advantages compared to systemic immunosuppression remains to be studied.

## 11. Future Directions

Advancing DC-targeted therapies in lung disease will require human-centered, spatially informed maps of subset and state heterogeneity. A priority is to resolve cDC2 and monocyte-derived DC subpopulations, their plasticity toward cDC1-like features, and the in situ tolerogenic programs that may be salvageable in chronic inflammation. Integrating single-cell multi-omics with spatial transcriptomics and multiplex imaging in well-phenotyped human tissues can reveal how niche signals and microanatomy—especially within tertiary lymphoid structures—shape DC functions and therapeutic responsiveness across COPD, IPF, asthma, and pulmonary hypertension. In parallel, mechanistic work must delineate when and how HIF-1α/mTOR-driven glycolysis versus AMPK-guided fatty-acid oxidation commits DCs to effector or tolerogenic trajectories. Combining metabolic flux approaches with pharmacologic and genetic perturbations will connect pathway control to antigen presentation, migration, and Treg induction, while prioritizing agents with existing clinical footprints (e.g., metformin, rapamycin, vitamin D) to hasten translational testing.

A second thrust is the intentional restoration of tolerance and checkpoint function. Ex vivo conditioning of tolerogenic DCs, alongside in vivo checkpoint-restoring and metabolic interventions, should be optimized for lung indications, with careful attention to durability of Treg expansion and safety in chronically inflamed tissues. Biomarker strategies, from PD-L1^+^ cDC1 frequencies in COPD to circulating DC numbers in IPF and chemokine signatures that forecast TLO activity, can guide patient selection and monitor response [138,148,169,170]. Because the journey of DCs is as consequential as their state, reprogramming migration deserves equal focus. Leveraging epigenetic control of Ccr7, PGE_2_-dependent transduction, and stromal chemokine fields to tune which DCs reach draining lymph nodes or iBALT/TLO niches could reduce pathogenic DC accumulation while preserving protective surveillance. Finally, DC–stromal–epithelial crosstalk must be dissected in systems that capture matrix and epithelial licensing—retinoic acid, GM-CSF, and versican processing to identify combinatorial strategies that target DCs alongside their “instructors” in the tissue microenvironment.

Translational success will hinge on aligning interventions with dominant DC pathology in each disease: dampening cDC2-driven Th2/Th17 programs in asthma and COPD, bolstering cDC1 protective circuits in pulmonary hypertension and fibrotic contexts, and choosing endpoints sensitive to inflammation, lymphoid neogenesis, vascular remodeling, and functional decline. Early-phase trials of metabolic conditioning, checkpoint restoration, or tolerogenic DC therapies that incorporate spatial and cellular biomarkers are within reach and, if timed to disease stage and delivered with tissue selectivity, may restore immune balance without compromising host defense.

## 12. Conclusions

This review highlights dendritic cells (DCs) as key orchestrators of lung immunity, converting local danger signals into specific adaptive responses while, in normal conditions, maintaining tolerance at mucosal surfaces constantly exposed to environmental stimuli. Evidence from diseases like COPD, IPF, asthma, and pulmonary hypertension emphasizes these roles: cDC2 expansion and activity correlate with vascular and airway damage, while cDC1 can sometimes offer protective effects depending on the context. This underscores the importance of developing approaches that target specific DC subsets, considering their timing and location. Overall, the field now views DCs not just as immune sentinels but as promising therapeutic targets: strategies such as restoring inhibitory checkpoints, reprogramming metabolism to promote tolerance, and modifying migratory pathways present feasible methods to adjust immune responses, avoiding the broad effects of global immunosuppression.

## Figures and Tables

**Table 1:** Dendritic cell subsets in the lung, their markers, functions, and context-dependent roles.

DC Sub-type	Key Markers (Human/Mouse)	Primary Functions	Context-Dependent or Paradoxical Roles
cDC1	Human: XCR1, CLEC9A; Mouse: CD103	Cross-presentation of antigens; CD8^+^ T-cell priming; Th1 polarization; antiviral and antitumor immunity	Loss exacerbates schistosomiasis-induced injury; reduced PD-L1^+^ cDC1 population in COPD correlates with rapid lung function decline and Th17-skewing
cDC2	Human: CD1c (BDCA-1), SIRPα; Mouse: CD11b, CD301b	MHC-II antigen presentation; strong inducers of Th2, Th17, and Tfh responses; key drivers of allergic and fibrotic pathways	Promote vascular remodeling in PH and COPD; depletion protects against hypoxia-induced PH; drive TLO formation via Tfh-like cell induction
pDCs	High type I/III interferon production phenotype	Production of type I/III IFNs during viral infection; bridge innate and adaptive immunity; promote tolerance via IL-10 and IDO	Under chronic inflammation may lose tolerogenic functions, contributing to dysregulated immune responses
moDCs	Derived from monocytes during inflammation	Amplify cytokine responses; promote T-helper polarization; sustain chronic inflammation in asthma and COPD	TLR3-activated moDCs can drive progression from acute viral infection to chronic lung disease; support airway remodeling

## Data Availability

No new data were created or analyzed in this study. Data sharing is not applicable to this article.
